# Effects of 100 IU versus 150 IU hCG supplementation on oocyte and embryo quality in patients aged ≥ 35 years: a propensity score-matched analysis

**DOI:** 10.7150/ijms.106965

**Published:** 2025-01-27

**Authors:** Nguyen Thi Lien Huong, Tran Thu Thuy, Phi Thi Tu Anh, Hoang Bao Long, Le Duc Thang, Vu Thi Ngoc, Le Quang Do, Nguyen Xuan Anh Duy, Bui Thi Hanh, Do Van Loi, Le Hoang

**Affiliations:** 1 Tam Anh General Hospital, Hanoi, Vietnam.; 2 College of Health Sciences, VinUniversity, Hanoi, Vietnam.; 3 Phenikaa University Hospital, Hanoi, Vietnam.

**Keywords:** controlled ovarian stimulation, human chorionic gonadotropin, *in vitro* fertilization, advanced maternal age, oocyte yield, embryo quality

## Abstract

**Background:** The optimal dose of human chorionic gonadotropin (hCG) supplementation during controlled ovarian stimulation (COS) remains controversial, particularly in women of advanced reproductive age.

**Objective:** To compare the efficacy and safety of 150 IU versus 100 IU daily hCG supplementation from stimulation day 6 in women aged ≥ 35 years undergoing *in vitro* fertilization/intracytoplasmic sperm injection (IVF/ICSI).

**Methods:** In this retrospective cohort study, we analyzed data from 438 patients aged ≥ 35 years undergoing IVF at a single center. Propensity score matching was performed at a 1:1 ratio, yielding 192 patients per group who received either 100 IU or 150 IU daily hCG supplementation from stimulation day 6 during GnRH antagonist cycles.

**Results:** After propensity score matching, baseline characteristics were comparable between groups. The 150 IU group yielded significantly more total oocytes (9.23±6.60 vs 7.79±5.52, p<0.001). However, the number of mature oocytes (6.25±5.12 vs 5.66±4.58) and day 3 embryos (5.22±4.62 vs 4.80±3.96) did not differ significantly between groups. Total FSH dose was similar (2664.84±141.91 vs 2666.02±142.51 IU, p=0.936). Only one case of moderate ovarian hyperstimulation syndrome occurred in the 150 IU group.

**Conclusions:** In women aged ≥ 35 years, while 150 IU daily hCG supplementation from stimulation day 6 safely increased total oocyte yield compared to 100 IU, it did not significantly improve mature oocyte numbers or embryo development.

## Introduction

*In vitro* fertilization (IVF) success rates decline substantially with advancing maternal age, particularly in women aged 35 years and older, primarily due to diminished ovarian reserve and reduced oocyte quality^1^,^2^. During controlled ovarian stimulation (COS) for IVF, adequate luteinizing hormone (LH) activity plays a crucial role in follicular development and oocyte maturation, with its importance potentially magnified in older women who often exhibit altered gonadotropin receptor expression and decreased ovarian sensitivity^3^,^4^.

The "two-cell, two-gonadotropin" theory emphasizes that both follicle-stimulating hormone (FSH) and LH are essential for optimal folliculogenesis. FSH promotes granulosa cell proliferation and aromatase activity, while LH stimulates theca cells to produce androgens for subsequent conversion to estradiol^5^. Recent evidence suggests that LH/human chorionic gonadotropin (hCG) signaling also directly influences oocyte quality through mitochondrial function and oxidative stress regulation pathways^6^. hCG shares structural homology with LH but exhibits distinct pharmacological advantages, including a longer half-life (24-36 hours versus 60 minutes) and stronger receptor binding affinity^7^. Recent molecular studies have shown that hCG induces more robust activation of ERK1/2 and AKT pathways crucial for follicular survival and steroidogenesis compared to LH^8^.

Several studies have investigated low-dose hCG supplementation during COS with promising results. Filicori *et al.* demonstrated that adding 200 IU/day of hCG from the mid-follicular phase could effectively complete follicular maturation while reducing FSH requirements^9^. The MERIT trial found that preparations containing hCG-derived LH activity resulted in higher top-quality embryo rates compared to recombinant FSH alone^10^. More recently, the MEGASET study showed improved ongoing pregnancy rates with hCG-supplemented protocols in high responders^11^.

Women aged ≥35 years, a subgroup known to benefit from LH activity supplementation during ovarian stimulation, may require different hCG doses due to age-related changes in ovarian function. These changes, including diminished androgen production, altered steroidogenesis, and reduced LH/hCG receptor expression, can lead to a suboptimal response to standard stimulation protocols^12^. The higher biological potency of hCG compared to LH may be particularly beneficial for women of advanced reproductive age due to its anti-apoptotic effects on follicles and ability to sustain androgen production. hCG has been shown to upregulate anti-apoptotic pathways in granulosa cells, including ERK1/2 and AKT signaling, which may rescue smaller follicles that would otherwise undergo atresia^8^. This follicular rescue effect could be especially important for older women with diminished ovarian reserve. Additionally, hCG's stronger LH receptor binding affinity compared to LH itself allows it to more potently stimulate theca cell androgen production^13^, which may help counteract the age-related decline in ovarian androgen levels that can impair folliculogenesis^14^.

The optimal dose of supplemental hCG remains debatable, particularly in women of advanced reproductive age. Previous studies have utilized doses ranging from 50 to 200 IU/day, with most protocols using either 100 or 150 IU/day^15^-^23^. A randomized controlled trial by Thuesen *et al.* suggested that 100 IU/day may represent the minimum effective dose, while doses up to 150 IU/day appeared safe and potentially more effective^16^. However, these studies included women across all age groups, and the specific requirements for older patients remain unclear.

Given the unique endocrine challenges faced by older women undergoing IVF, such as altered gonadotropin receptor expression and reduced ovarian sensitivity^12^, age-specific investigations are needed to determine the most effective and safe hCG dosing strategies for this subgroup. Therefore, we conducted this propensity score-matched retrospective cohort study to compare the effects of 100 IU versus 150 IU daily hCG supplementation from stimulation day 6 on oocyte and embryo outcomes in women aged ≥35 years undergoing IVF. Our findings may help establish evidence-based recommendations for hCG supplementation protocols in this challenging patient population.

## Materials and Methods

### Study Design and Population

This retrospective cohort study was conducted at our academic fertility center between January 2020 and September 2023. The study protocol was approved by the Institutional Review Board (IRB.TAHN.024/03). Patient data was collected from two distinct time periods corresponding to different hCG supplementation protocols: Period 1 (January 2020-July 2022) when 100 IU hCG was used and Period 2 (July 2022-September 2023) when 150 IU hCG was administered.

Eligible patients were women aged ≥35 years undergoing IVF/ICSI, using GnRH antagonist protocol and receiving hCG supplementation from stimulation day 6. Exclusion criteria were endometrioma, prior ovarian surgery, severe male factor requiring surgical sperm retrieval or severe oligoasthenoteratozoospermia, and parental chromosomal abnormalities.

### Ovarian Stimulation Protocol

Individualized starting doses of recombinant FSH (Follitrope, LG Chem, Korea) ranging from 225-300 IU were administered based on patient characteristics including age, body mass index (BMI), anti-Müllerian hormone (AMH) level and previous response. GnRH antagonist cetrorelix (Cetrotide 0.25mg, Merck Serono, Germany) was started on stimulation day 6 and continued until trigger. In addition to the GnRH antagonist, patients received daily supplementation with either 100 IU (Period 1) or 150 IU (Period 2) of highly purified hCG (Pregnyl, MSD, Netherlands or IVF-C, LG Chem, Korea). When at least two follicles reached 17 mm in mean diameter, final oocyte maturation was triggered with 5,000 IU hCG (IVF-C, LG Chem, Korea) and 0.2 mg triptorelin acetate (Fertipeptil, Ferring, Germany). Transvaginal ultrasound-guided oocyte retrieval was performed 35-36 hours later under intravenous sedation.

### Laboratory Procedures

Retrieved oocytes were assessed for nuclear maturity after denudation. Intracytoplasmic sperm injection (ICSI) was performed on mature oocytes (MII) that exhibited the first polar body. Fertilization was confirmed 16-18 hours post-ICSI by the presence of two pronuclei. Embryos were cultured in sequential media (Vitrolife, Sweden) or single-step medium (Irvine Scientific, United States), under standard conditions (37°C, 6% CO_2_, 5% O_2_). Cleavage embryo morphology was assessed daily according to consensus scoring criteria based on cell number, symmetry, fragmentation and multinucleation^24^. Good-quality day 3 embryos were defined as those with 6-8 cells, <20% fragmentation, and stage-appropriate cell size. The classification of obtained blastocysts was based on Gardner and Schoolcraft's criteria, which consider blastocoel expansion, as well as the morphology of the trophoblast and inner cell mass. Blastocysts with grades of 3BB or higher were identified as being of good quality^25^.

### Outcome Measures and Definitions

The primary outcomes were number of MII oocytes retrieved and number of day 3 embryos obtained. Secondary outcomes included total FSH dose, estradiol level on trigger day, number of follicles 14-24 mm on trigger day, number of good-quality day 3 embryos and blastocysts, and incidence of ovarian hyperstimulation syndrome (OHSS). OHSS was classified according to the American Society for Reproductive Medicine guidelines^26^.

### Statistical Analysis

The sample size calculation was based on previously published data from Thuesen *et al.*, which indicated a mean number of oocytes retrieved of 9.2 ± 4.2 in the 100 IU group and 11.3 ± 5.7 in the 150 IU group^16^. We assumed a difference of 2.1 in the mean number of oocytes retrieved between the two groups. Setting the two-tailed α level at 0.05 and the power (1-β) at 80%, a minimum of 90 cycles were required for each group. Considering a potential dropout rate of 10%, we aimed to include at least 100 cycles in each group.

Continuous variables were tested for normality using the Shapiro-Wilk test and presented as mean ± standard deviation or median (interquartile range). Categorical variables were presented as number (percentage). Between-group comparisons were conducted using Student's t-test or Mann-Whitney U test for continuous variables and chi-square or Fisher's exact test for categorical variables, as appropriate.

**Figure 1:**
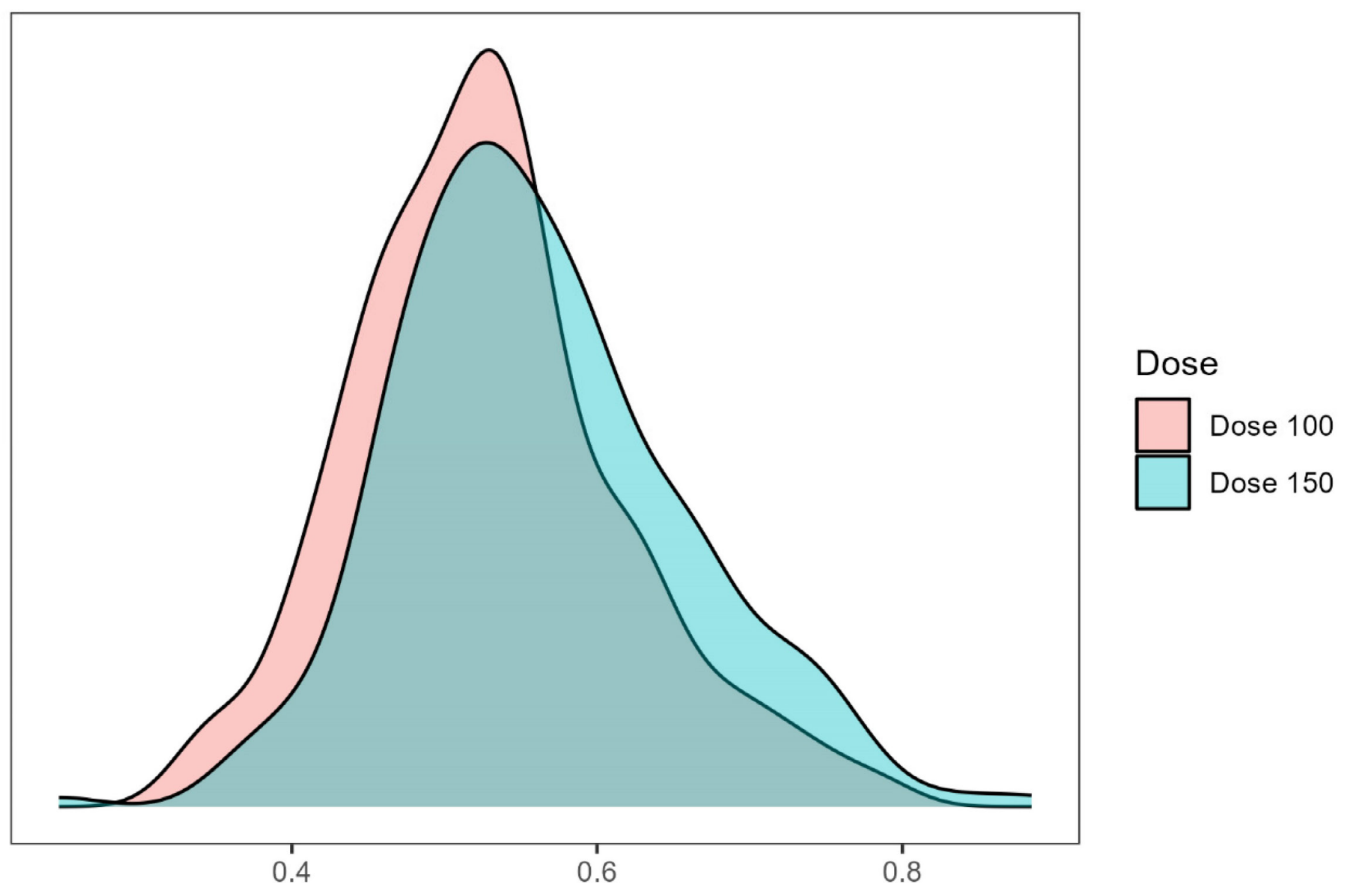
Kernel density estimate plot of propensity scores. The propensity scores (probability of being treated with dose 100) were constructed by a multiple logistic regression model. The distribution of propensity scores between the two protocols showed adequate overlap (common support) to conduct a propensity score analysis on the entire study population.

**Figure 2:**
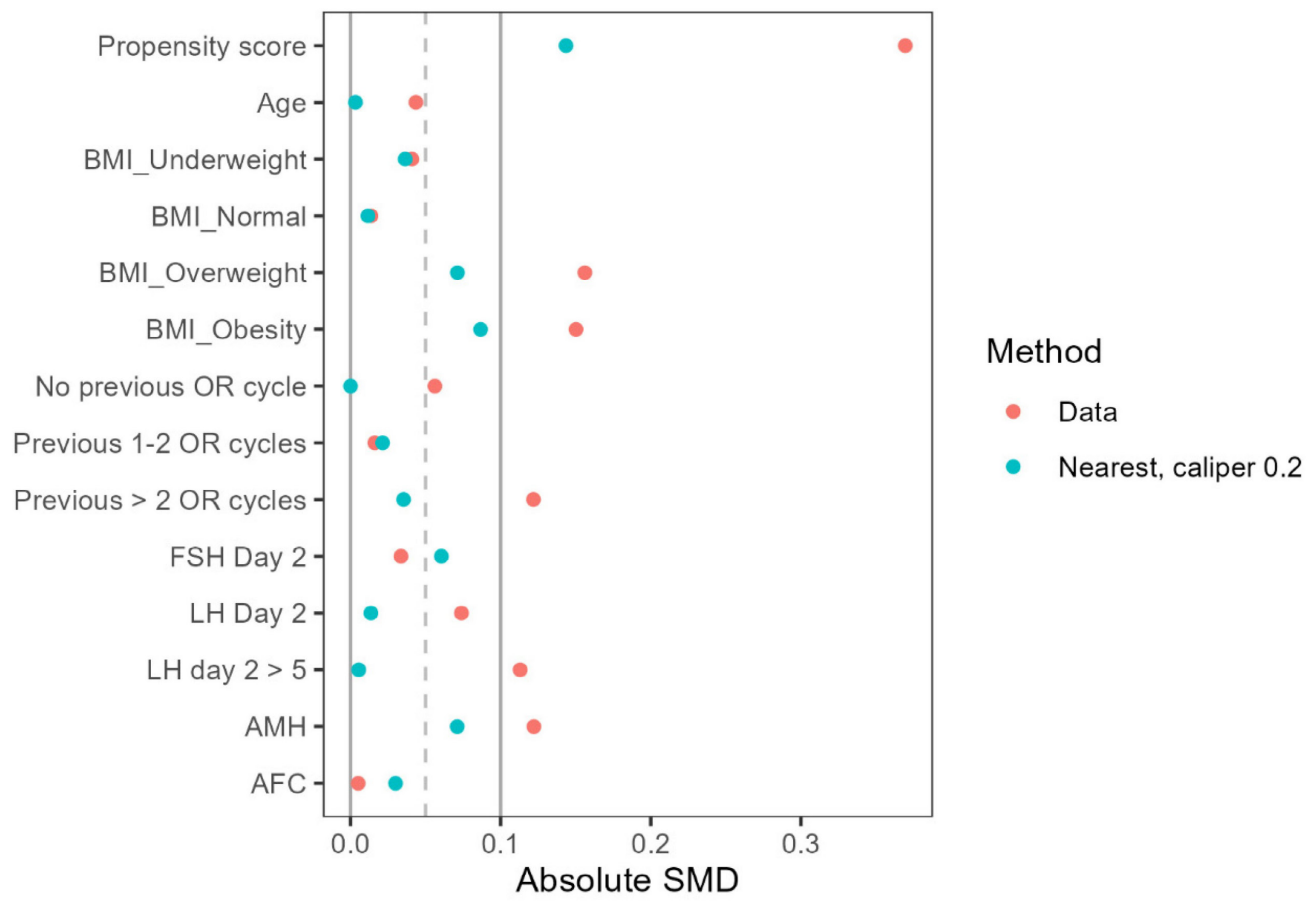
A Love plot evaluating the balance after two propensity score matching methods.

To minimize confounding bias, propensity score matching was performed using a 1:1 nearest neighbor matching algorithm with a caliper of 0.2 and no replacement. Variables included in the propensity score model were age, BMI, previous IVF attempts, infertility factors, baseline FSH, LH, AMH, and antral follicle count (AFC). The kernel density plot (Figure 1) and Love plot (Figure 2) are presented to visually illustrate the propensity score distribution and covariate balance achieved after matching.

The effect of hCG dose on primary and secondary outcomes was evaluated using negative binomial regression models, both unadjusted and adjusted using the propensity score method. Results were expressed as mean ratios with 95% confidence intervals (CIs). For outcomes only available in a subset of patients (blastocyst parameters), the number of patients with data in each group was reported.

All statistical tests were two-tailed, with p-values <0.05 considered statistically significant. Statistical analysis was performed using R version 4.2.1.

## Results

After propensity score matching, 384 patients were included in the final analysis (192 per group). Baseline characteristics were comparable between the 100 IU and 150 IU hCG groups (Table 1). The mean age (38.88 ± 3.14 vs 38.89 ± 3.28 years, p=0.975), age distribution (64.6% vs 62.5% aged 35-39 years), BMI (21.69 ± 1.97 vs 21.81 ± 2.19 kg/m^2^), baseline FSH (9.04 ± 3.87 vs 8.80 ± 3.80 mIU/mL), AMH (1.94 ± 1.43 vs 1.84 ± 1.36 ng/mL), and AFC (10.89 ± 7.13 vs 10.68 ± 6.25) did not differ significantly between groups. Compared to the population before matching, the characteristics in the population after matching were very similar.

The duration of ovarian stimulation was similar between groups (10.18 ± 0.63 days), with comparable total FSH dose (2664.84 ± 141.91 vs 2666.02 ± 142.51 IU, p=0.936). End-of-stimulation hormone profiles showed higher levels in the 150 IU group for both estradiol (3281.35 ± 2504.58 vs 3000.74 ± 2336.16 pg/mL, p=0.257) and progesterone (0.95 ± 0.90 vs 0.73 ± 0.51 ng/mL, p=0.004). The number of preovulatory follicles (14-24mm) on trigger day was numerically higher in the 150 IU group (8.01 ± 5.42 vs 6.74 ± 5.42, p=0.141).

The total number of oocytes retrieved was significantly higher in the 150 IU group compared with the 100 IU group (9.23 ± 6.60 vs 7.79 ± 5.52, p<0.001), a difference that persisted after adjusting for potential confounders (adjusted mean ratio - aMR 0.87, 95% CI 0.79-0.96) (Table 3). However, the number of mature MII oocytes did not differ significantly between groups in adjusted analyses (6.25 ± 5.12 vs 5.66 ± 4.58, aMR 0.93, 95% CI 0.82-1.06). The number of day 3 cleavage stage embryos was higher in the 150 IU group (5.22 ± 4.62 vs 4.80 ± 3.96), but the difference did not reach statistical significance (aMR 0.97, 95% CI 0.84-1.11). The proportion of good-quality embryos on day 3 was also similar (3.65 ± 3.60 vs 3.35 ± 3.23, aMR 0.96, 95% CI 0.80-1.14). For the subgroup of patients having blastocyst culture, the higher hCG dose did not significantly impact total blastocyst number (3.89 ± 3.59 vs 3.72 ± 2.92, aMR 0.93, 95% CI 0.77-1.13) or good quality blastocyst formation (2.23 ± 2.62 vs 2.21 ± 2.10, aMR 0.95, 95% CI 0.72-1.24).

Regarding safety, only one case of moderate OHSS (0.5%) occurred in the 150 IU group, with none in the 100 IU group.

## Discussion

In this propensity score-matched retrospective cohort study, we found that increasing daily hCG supplementation from 100 IU to 150 IU starting from stimulation day 6 in GnRH antagonist cycles significantly increased the total number of oocytes retrieved in women ≥35 years old undergoing IVF/ICSI. While mature oocyte and day 3 embryo yields also favored the 150 IU group, these differences did not reach statistical significance after adjusting for confounders. Embryo quality parameters and blastocyst development were comparable between the two doses. Only one case of moderate OHSS occurred with 150 IU hCG, suggesting a favorable safety profile in this population.

Our observation that 150 IU hCG produced more oocytes than 100 IU (9.23 vs 7.79, p<0.001) contrasts with some previous findings. Madani *et al.* compared 100 IU versus 200 IU hCG supplementation in poor responders and found comparable outcomes between doses^17^. However, their study population differed from ours in including only poor responders. More relevant to our age-focused analysis, Gomaa *et al.* demonstrated that low-dose hCG supplementation particularly benefited women over 40 years, though they did not directly compare different hCG doses^19^. The discrepancy between the increased oocyte yield and lack of improvement in mature oocyte and embryo numbers with 150 IU hCG may be explained by a potential ceiling effect, where higher hCG doses can support the growth and maturation of a larger cohort of follicles up to a certain stage, but may not necessarily enhance their final acquisition of developmental competence. The increased oocyte yield in the 150 IU group may have been primarily driven by the improved survival and growth of smaller antral follicles, which are more sensitive to the anti-apoptotic effects of hCG^27^. These rescued follicles, while contributing to the total oocyte count, might not have had sufficient time or optimal conditions for complete oocyte maturation. Further research is needed to elucidate the molecular mechanisms governing the acquisition of oocyte developmental potential in the context of hCG supplementation.

The estradiol levels observed in the 150 IU group (3281 vs 3001 pg/mL, p=0.257) did not differ significantly from the 100 IU group. This observation aligns with Thuesen *et al.*'s dose-response study which demonstrated that serum estradiol reached a plateau with hCG doses above 100 IU/day, despite continuing increases in androgen production at higher doses^16^. The lack of significantly higher estradiol with 150 IU hCG supports the concept of an estradiol ceiling effect, likely due to saturation of aromatase activity in converting androgens to estrogens, as proposed by Smitz and Platteau^8^.

The significantly higher progesterone levels observed in the 150 IU hCG group (0.95 vs 0.73 ng/mL, p=0.004) warrant further discussion. This finding is consistent with the known dose-dependent effect of hCG on ovarian steroidogenesis, particularly its ability to stimulate progesterone production by luteinized granulosa cells^16^. The higher progesterone levels in the 150 IU group likely reflect the increased steroidogenic activity of a larger cohort of hCG-responsive follicles, as evidenced by the significantly higher oocyte yield compared to the 100 IU group. While elevated progesterone levels have been associated with impaired endometrial receptivity in fresh embryo transfer cycles, it is important to note that the mean progesterone level in our 150 IU group (0.95 ng/mL) remained below the threshold of 1.5 ng/mL, above which negative effects on clinical outcomes have been consistently reported^28^. Furthermore, our universal application of a freeze-all strategy mitigates any potential detrimental impact of elevated progesterone on endometrial receptivity, as the endometrium is not exposed to the supraphysiologic hormonal milieu of ovarian stimulation during the subsequent frozen embryo transfer cycle^29^.

While we did not directly measure androgen levels, the higher progesterone levels may indirectly reflect increased androgen substrate availability^30^. Elevated intraovarian androgens have been proposed to enhance follicular FSH sensitivity and promote follicular survival^14^. However, excessive androgens may also impair oocyte quality and embryo development^31^. The lack of improvement in embryo quality parameters in our study despite higher oocyte yields with 150 IU hCG suggests that any potential benefits of increased androgens were outweighed by other factors governing oocyte competence.

Regarding safety, our finding of only one moderate OHSS case with 150 IU hCG compares favorably with previous studies. Kosmas *et al.*'s meta-analysis found that low-dose hCG supplementation (50-200 IU) actually reduced OHSS risk compared to conventional protocols^32^. Although the mechanism remains unclear, it is possible that hCG supports the survival and growth of smaller follicles, allowing them to develop into larger follicles, thereby reducing the number of smaller follicles associated with OHSS risk.

Our findings underscore the importance of prioritizing oocyte quality over quantity in the pursuit of optimal IVF outcomes. While maximizing oocyte yield may be an attractive goal, the lack of a corresponding improvement in embryo quality suggests that this approach may not be universally beneficial. This discrepancy raises questions about the cost-effectiveness of using higher hCG doses to maximize oocyte yield, particularly in patients planning for oocytes cryopreservation. In clinical practice, the decision to use a higher hCG dose should be weighed against the potential risks and benefits for each patient. For those with diminished ovarian reserve or a history of suboptimal response, a modest increase in oocyte yield may prove advantageous, providing more opportunities for embryo selection. However, this potential benefit must be balanced against the lack of improvement in embryo quality and the increased cost associated with higher gonadotropin consumption.

The key strengths of our study include the use of propensity score matching to minimize confounding bias, the large sample size, and standardized protocols. The single-center setting also reduces heterogeneity in clinical and laboratory practices. However, this study has several limitations. The retrospective design and nonrandomized allocation of patients to hCG dosing groups may have introduced bias, although this was mitigated by propensity score matching. The lack of significant differences in mature oocyte and embryo numbers between the two groups may have been influenced by the sample size, which was primarily powered to detect changes in total oocyte yield. Larger studies are needed to further elucidate the impact of hCG dosing on these key outcomes and to assess the potential effects on clinical pregnancy and live birth rates.

In conclusion, this propensity-matched retrospective study suggests that while increasing daily hCG supplementation from 100 IU to 150 IU from stimulation day 6 safely improves total oocyte yield in IVF/ICSI patients ≥35 years old, it may not significantly enhance the more clinically relevant endpoints of mature oocyte yield and embryo outcomes. These findings highlight the need to carefully consider the cost-effectiveness and individualized risk-benefit balance when selecting hCG doses for ovarian stimulation in older women. Further research is needed to evaluate the impact of late follicular hCG dosing on live birth rates and elucidate the molecular mechanisms governing oocyte maturation.

## Figures and Tables

**Table 1:** Baseline characteristics of study patients

Characteristic	Before matching			After matching		
	hCG 100 IU (n = 199)	hCG 150 IU (n = 239)	p-value	hCG 100 IU (n = 192)	hCG 150 IU (n = 192)	p-value
Age (years)	38.87 (3.14)	38.74 (3.17)	0.649	38.88 (3.14)	38.89 (3.28)	0.975
Age group, n (%)			1.000			0.750
35 - 39 years	128 (64.3)	153 (64.0)		124 (64.6)	120 (62.5)	
> 39 years	71 (35.7)	86 (36.0)		68 (35.4)	72 (37.5)	
BMI (kg/m2)	21.73 (1.95)	21.92 (2.26)	0.349	21.69 (1.97)	21.81 (2.19)	0.567
Infertility duration (years)	4.15 (4.99)	3.40 (4.36)	0.095	4.21 (5.05)	3.15 (4.22)	0.027
Previous IVF attempts, n (%)			0.380			0.914
0 cycle	108 (54.3)	123 (51.5)		103 (53.6)	103 (53.6)	
1-2 cycles	79 (39.7)	93 (38.9)		77 (40.1)	75 (39.1)	
> 2 cycles	12 (6.0)	23 (9.6)		12 (6.2)	14 (7.3)	
Basal FSH (mIU/mL)	8.94 (3.84)	8.80 (3.96)	0.723	9.04 (3.87)	8.80 (3.80)	0.541
Basal LH (mIU/mL)	5.96 (2.43)	6.15 (2.53)	0.433	5.99 (2.46)	5.96 (2.11)	0.884
AMH (ng/mL)	2.03 (1.52)	1.86 (1.45)	0.213	1.94 (1.43)	1.84 (1.36)	0.470
AFC	11.03 (7.11)	11.00 (6.78)	0.959	10.89 (7.13)	10.68 (6.25)	0.767

Data expressed as mean (SD) unless otherwise specified. BMI, body mass index; IVF, *in vitro* fertilization; FSH, follicle-stimulating hormone; LH, luteinizing hormone; AMH, anti-Müllerian hormone; AFC, antral follicle count.

**Table 2:** Characteristics of cycle parameters after matching

Characteristic	hCG 100 IU (n = 192)	hCG 150 IU (n = 192)	p-value
Length of stimulation (days)	10.18 (0.63)	10.18 (0.63)	0.936
Total FSH dose (IU)	2664.84 (141.91)	2666.02 (142.51)	0.936
Estradiol level on trigger day (pg/mL)	3000.74 (2336.16)	3281.35 (2504.58)	0.257
Progesterone level on trigger day (ng/mL)	0.73 (0.51)	0.95 (0.90)	0.004
Number of follicles 14-24 mm	6.74 (5.42)	8.01 (5.42)	0.141

**Table 3:** Comparison of outcomes between two groups

Variables	hCG 100 IU (n = 192)	hCG 150 IU (n = 192)	Unadjusted mean ratio (95% CI)	Adjusted mean ratio (95% CI)
Number of oocytes retrieved	7.79 (5.52)	9.23 (6.60)	0.84 (0.73, 0.97)	0.87 (0.79, 0.96)
Number of mature oocytes	5.66 (4.58)	6.25 (5.12)	0.90 (0.77, 1.07)	0.93 (0.82, 1.06)
Number of cleavage embryos	4.80 (3.96)	5.22 (4.62)	0.92 (0.77, 1.09)	0.97 (0.84, 1.11)
Number of good cleavage embryos	3.35 (3.23)	3.65 (3.60)	0.92 (0.75, 1.12)	0.96 (0.80, 1.14)
Number of blastocysts^a^	3.72 (2.92)	3.89 (3.59)	0.96 (0.77, 1.18)	0.93 (0.77, 1.13)
Number of good blastocyst^a^	2.21 (2.10)	2.23 (2.62)	0.99 (0.76, 1.29)	0.95 (0.72, 1.24)

Data expressed as mean (SD). CI, confidence interval. ^a^ Data analyzed in 113 patients (hCG 100 IU group) and 140 patients (hCG 150 IU group) who cultured all embryos to blastocyst stage.

## Abbreviations

AFC: antral follicle count
AMH: anti-Müllerian hormone
aMR: adjusted mean ratio
BMI: body mass index
CI: confidence interval
COS: controlled ovarian stimulation
FSH: follicle-stimulating hormone
GnRH: gonadotropin-releasing hormone
hCG: human chorionic gonadotropin
ICSI: intracytoplasmic sperm injection
IU: international unit
IVF: *in vitro* fertilization
LH: luteinizing hormone
OHSS: ovarian hyperstimulation syndrome
